# Identifying Barriers to Patient Acceptance of Active Surveillance: Content Analysis of Online Patient Communications

**DOI:** 10.1371/journal.pone.0068563

**Published:** 2013-09-11

**Authors:** Mark V. Mishra, Michele Bennett, Armon Vincent, Olivia T. Lee, Costas D. Lallas, Edouard J. Trabulsi, Leonard G. Gomella, Adam P. Dicker, Timothy N. Showalter

**Affiliations:** 1 Department of Radiation Oncology, University of Maryland, Baltimore, Maryland, United States of America; 2 Wool Laboratories Inc., Wayne, Pennsylvania, United States of America; 3 Department of Urology, University of California Davis, Sacramento, California, United States of America; 4 Department of Urology, Thomas Jefferson University, Philadelphia, Pennsylvania, United States of America; 5 Department of Radiation Oncology, University of Virginia, Charlottesville, Virginia, United States of America; University of Vermont, United States of America

## Abstract

**Objectives:**

Qualitative research aimed at identifying patient acceptance of active surveillance (AS) has been identified as a public health research priority. The primary objective of this study was to determine if analysis of a large-sample of anonymous internet conversations (ICs) could be utilized to identify unmet public needs regarding AS.

**Methods:**

English-language ICs regarding prostate cancer (PC) treatment with AS from 2002–12 were identified using a novel internet search methodology. Web spiders were developed to mine, aggregate, and analyze content from the world-wide-web for ICs centered on AS. Collection of ICs was not restricted to any specific geographic region of origin. NLP was used to evaluate content and perform a sentiment analysis. Conversations were scored as positive, negative, or neutral. A sentiment index (SI) was subsequently calculated according to the following formula to compare temporal trends in public sentiment towards AS: [(# Positive IC/#Total IC) - (#Negative IC/#Total IC) x 100].

**Results:**

A total of 464 ICs were identified. Sentiment increased from -13 to +2 over the study period. The increase sentiment has been driven by increased patient emphasis on quality-of-life factors and endorsement of AS by national medical organizations. Unmet needs identified in these ICs include: a gap between quantitative data regarding long-term outcomes with AS vs. conventional treatments, desire for treatment information from an unbiased specialist, and absence of public role models managed with AS.

**Conclusions:**

This study demonstrates the potential utility of online patient communications to provide insight into patient preferences and decision-making. Based on our findings, we recommend that multidisciplinary clinics consider including an unbiased specialist to present treatment options and that future decision tools for AS include quantitative data regarding outcomes after AS.

## Introduction

Qualitative research aimed at identifying patient acceptance of active surveillance (AS) has been identified as a public health research priority [1]. The currently available literature on AS decision-making has identified the influence of several patient- and provider-specific factors impacting utilization of AS for low-risk PC [2,3]. Although these studies provide a useful framework for understanding the issue, they are limited by small sample sizes of patients, typically from a single-institution, and the semi-structured nature of the interviews conducted by investigators to obtain information.

Given the growing role of the participatory web today [4], an increasing number of patients now utilize online support groups and social media websites to discuss and express opinions about different treatment options [5]. The unstructured nature of such communications has traditionally made it difficult to analyze such data. However, recent advances in computer technology [4,6] have now made it feasible to analyze patient-generated internet content for biomedical research purposes [5,7]. This presents a rich and powerful resource for healthcare providers to better understand public perception and sentiment, and also represents a novel methodology for furthering the patient-centered agenda of the Institute of Medicine [7,8].

In this study, we utilized commercially-available software (Wool Labs., Inc) to mine, aggregate, and analyze patient-generated internet content on the subject AS. The primary objective of this study was to determine if analysis of a large-sample of anonymous internet conversations (ICs) could be utilized to identify unmet public needs regarding AS.

## Materials and Methods

### Data Collection

After obtaining exemption status from the Thomas Jefferson University Institutional Review Board, publicly-available, ICs regarding PC treatment with AS or WW from 2002–2012 were identified using a commercially-available internet search software (Wool Labs Inc., Wayne Pennsylvania). Web spiders were developed to mine and aggregate content from the world-wide-web for ICs centered on prostate cancer or prostate adenocarcinoma. This process was fully automated and revealed 3499 ICs. All websites with ICs were screened programmatically to remove any spam or not-on-topic ICs and websites. A total of 34 distinct websites were utilized for data extraction and are listed in Table 1.

**Table 1:** Websites used to collect internet conversations.

http://www.medkb.com/
http://www.orlandoprostatecancer.com/
http://blogs.wsj.com/health/
http://forum.urologychannel.com/
http://forums.corvetteforum.com/
http://glasshospital.com/
http://health.usnews.com/
http://itsaguythingblog.wordpress.com/
http://legalmedicine.blogspot.com
http://neilbaum.wordpress.com
http://pcneedtogo.blogspot.com/
http://prostatecancerblog.net/
http://prostatecancerinfolink.net/
http://prostatecancerinfolink.net/
http://scienceblog.com/
http://theprostatedecision.wordpress.com/
http://twitter.com
http://vets.yuku.com/
http://well.blogs.nytimes.com/
http://www.buzzmachine.com/
http://www.cancercompass.com/
http://www.cancerforums.net/
http://www.drwalt.com/
http://www.fodors.com/
http://www.healingwell.com/
http://www.healthboards.com/
http://www.health-forums.com/
http://www.healthnewsreview.org/
http://www.healthnewsreview.org/
http://www.inspire.com/
http://www.medhelp.org/
http://www.npr.org/
http://www.revolutionhealth.com/
http://www.watchwait.com/

Conversations were then limited to those on the subject of AS, WW, ‘expectant management’, or ‘wait-and-see’, leaving 464 ICs for analysis. For the purposes of the present analysis, an IC was defined by at least one message or reply to published web content, and each IC typically consists of a dialogue of multiple entries.

Collection of ICs was not restricted to any specific geographic region of origin, but only English-language ICs were included in the present analysis. Counts of conversation by year, subtopic groupings and sentiment terminology were extracted. The date of ICs was determined based upon the timestamp of the IC listed on the website. Sample ICs are shown in Tables 2-5. The full corpus of ICs is not displayed in order to comply with copyright right laws.

**Table 2:** Representative Patient Comments: 2000-2005.

**Representative Patient Comments: 2000-2005**	**Sentiment**
*I’m not a fan of WW, unless you happen to have some other more serious health issues... The idea of having PSA tests every two months and twice yearly biopsies is not appealing to me.*	negative
*I had a PSA of 6.35…. and Gleason of 5 or 6. I flat out asked [my doctor*]* if I did watchful waiting, how long would I live? After reviewing my records for a couple of minutes, he said that you will not see 70 and your last three years will be pain.*	negative
If a man has low grade disease and is willing to get it accurately staged and then methodically follow it, and if his personality is such that he can live comfortably knowing that there is cancer inside of him, then maybe WW / AS is an option. .	positive
*To me, doing nothing is NOT an option. It will be one of the biggest mistakes that could happen and you only pay for it with your life.*	negative

**Table 3:** Representative Patient Comments: 2006-2007.

**Representative Patient Comments: 2006-2007**	**Sentiment**
*Because I was foolish, I ended up in a "watchful waiting" category for close to four years before I was treated.*	negative
*No prudent physician would even suggest a young man wait for very long. If the [doctor*]* can, and surgery is your choice, ask about seminal vesicle tip sparing as well as nerve sparing.*	negative
*I am doing active surveillance. . I really, really refuse at this point to do one of the Big 3... Would someone please share with me the company websites, email… [for*]* obtaining any the natural supplements*	positive
*We have been involved in numerous medical malpractice cases involving the failure to timely diagnose and treat [PC*]*… The first line of defense for these doctors when confronted with a medical negligence case is to assert that an elevated PSA level does not require any medical treatment – only “watchful waiting” to see if the PSA continues to rise or if other indicators of cancer are present. Now, a recent study published in the Journal of the American Medical Association (JAMA*)* seems to refute the watchful waiting approach and encourages patients to consider treatment options at the earliest opportunity.*	negative

**Table 4:** Representative Patient Comments: 2008-2009.

**Representative Patient Comments: 2008-2009**	**Sentiment**
*In 2008, if a man has a Gleason sum of 6 or less, and a PSA doubling time of more than two years, it seems obvious to me he should choose active surveillance and hope for technological developments while remaining vigilant for unusual developments in his own particular case. Most men do not realize that prostatectomy has a [high*]* likelihood of impaired sexual function. .*	positive
*Prostate cancer treatment should be determined by many existing circumstances: including the patient’s age, sex life, and marital status. After a radical prostatectomy often, [a patient’s*]* patience sex life is over.*	neutral
*I have a [friend*]*…. who had 1% cancer in his prostate. Gleason 6. He has terrible ED and probably should have done watchful waiting for years to come. My heart breaks for him.*	positive
*This AUA statement should strengthen doctors’ confidence in Active Surveillance for truly low-risk men, and I hope it will add to knowledge in our own survivor community*	positive
*It is important for people to recognize that prostate cancer is a complex disease. There isn’t a single prescription which applies to all men… He has to consider the specifics of his diagnosis, as well as what risks he is willing to take and what he considers most important.*	positive
*The problem with AS as I see it… [is that*]* it is impossible to know exactly when the tumor progresses and/or becomes more aggressive.*	negative
A case can be made for [active surveillance] statistically, but I wonder how many doctors defer their own treatment when diagnosed with prostate cancer.	negative
*I can’t understand risking having a more advanced cancer in his 70s vs. taking care of it all now. Women can’t really relate to this. I have many women friends who have taken their breasts off rather than risk further breast cancers. I’d rather have my husband into his 80s and 90s without erections than lose him or see him go through horrible treatments.*	negative

**Table 5:** Representative Patient Comments: 2010-2012.

**Representative Patient Comments: 2010-2012**	**Sentiment**
*One simply needs to look at the advertisements for different treatment that are presented to the public to see the source of the misconceptions on benefits of treatment. The “cure rate” is featured prominently in many… without clarification.*	positive
*We still need a new tool to find out if these cancers are low risk or not. The PSA and * *Gleasonscore* * are famous for inaccuracy.*	negative
*Perhaps we need prostate cancer active “surveillists,” a sub-specialty committed to minimizing the risk of inaccurately diagnosing an indolent prostate cancer as prostate cancer that requires treatment, managing cases of low-risk, low-grade prostate cancer to minimize the risk of too early and excessive treatment, and providing pre- and post-medical management and educational support to minimize quality-of-life-destroying side effects for men whose prostate cancer does progress to the point of productively and rationally requiring invasive, radical intervention.*	positive
*... there needs to more and better education for patients and families of those diagnosed with prostate cancer about active surveillance. I participate in several on-line prostate cancer support groups. The suggestion of active surveillance is almost seen as insulting by many members of the group.*	positive
*Doctors are paid for doing procedures. That is what the "P" in CPT (the billing code*)* stands for. If a patient wants to get unbiased information, he’s got to seek it out. I think a series of videos, podcasts, etc., can provide this better than harried doctors who aren’t paid for the substantial time it takes to educate, and who might very well have a bias in doing a type of procedure in which they have invested much time and perhaps quite a deal of money.*	positive
*… repeated biopsies do cause a lot of trauma to the prostate and an increased risk for other infections. I find the prospect of regular biopsies stressful and they also have an impact on one’s sex life.*	negative
*I believe the key to increasing the use of active surveillance is to adopt a patient-centered care approach. First determine what the patient’s life goals are. Provide all the necessary information for the patient to make a decision about treatment options to meet those goals. Have shared decision-making on how to attain those goals. The key is to put the patient in the center of the process.*	positive
*I have been on [AS*]* for two years. [My doctor*]* told me I may live for 15 years with no problems, until a recent biopsy [indicated*]* I needed treatment. So I just had robotic surgery and am now left with a 3mm positive margin. If I had taken action straight away, I would have had a complete removal of the cancer. In my view had I the choice again I would not have worried about the side effects. .*	negative

## Data Analysis

After screening, anonymized cognitive data from IC were analyzed using the Wool Labs© (Wool Labs, Inc., Wayne PA) natural language processing (NLP) processor. The NLP processor utilizes a logic-based methodology, which involves both inductive (Observation - Pattern - Tentative Hypothesis – Theory) and deductive (Theory - Hypothesis - Observation - Confirmation) approaches [9].

A NLP-mediated sentiment analysis was subsequently performed to determine sentiment towards AS in each IC, and to compare temporal trends in public sentiment towards AS. A three-way task model was utilized to classify sentiment into positive, negative or neutral classes [10].

The NLP-mediated analysis was performed utilizing a machine-learning approach. Briefly, the NLP processor software assigns sentiment based upon an internal data dictionary of basic language constructs as well as common healthcare terminology. Additionally, an investigator (MB) reviewed 200 ICs and scored each IC as positive, negative, or neutral. A machine-learning approach was then applied to force the NLP processor to mimic the results of the human sentiment analysis. Following this process, the NLP processor was applied to the entire cohort of ICs (n=464). All ICs were subsequently reviewed by investigators (MB, MM, and TS) to verify the accuracy of the sentiment analysis. In total, 0.5% of ICs had a sentiment score that was modified following investigator review.

A sentiment index (SI) was subsequently calculated according to the following formula in order determine temporal trends in patient sentiment: SI = [(# Positive IC/#Total IC) – (#Negative IC/#Total IC) x 100].

The SI may range from -100 to +100.

## Results

**Figure 1:**
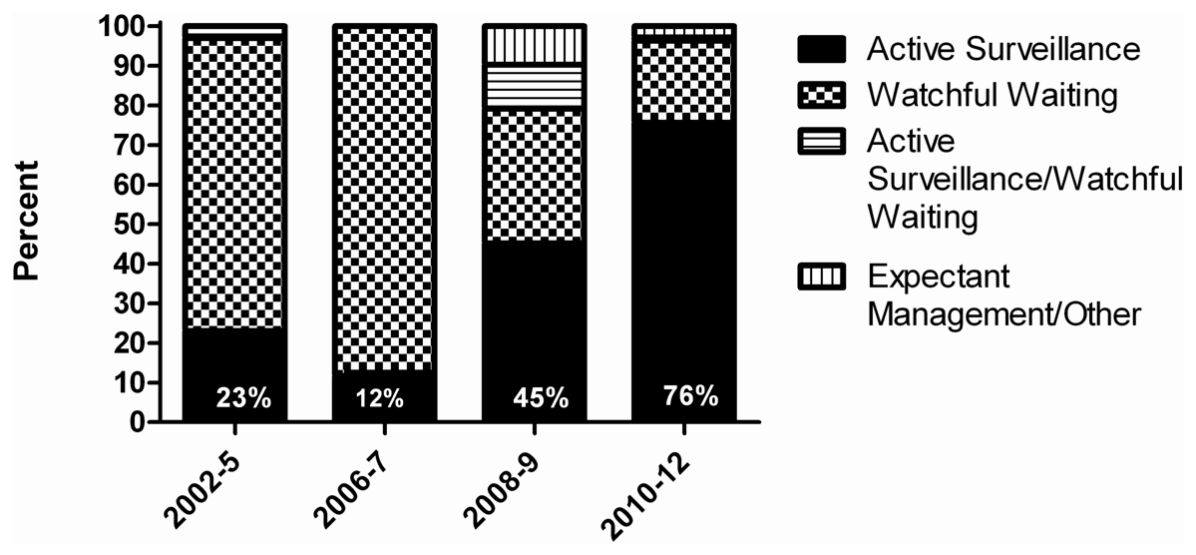
Use of terminology in internet conversations to describe observational strategies for prostate cancer, according to study periods.

A total of 464 anonymous ICs from 2002–2012 were identified that involved PC treatment with AS, WW or expectant management. The terminology used in ICs prior to 2007 was most commonly WW (range of annual percentages: 74-88%). Beginning in 2008-9, IC terminology shifts to more commonly mention AS (45%). From 2010–2012, AS becomes the most commonly used terminology in ICs (76%). Terminology use stratified by year of IC is shown in Figure 1.

**Figure 2:**
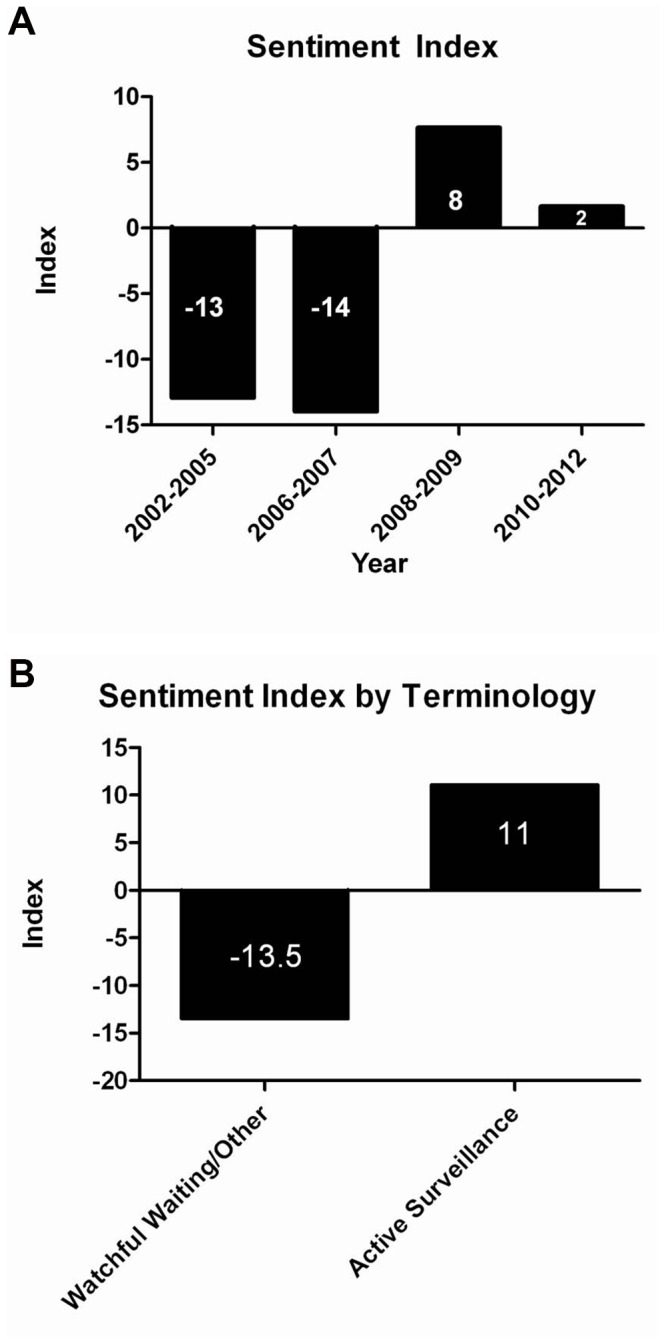
Sentiment Index of internet conversations stratified by year (A) and terminology (B).

In total, 30.2% of ICs were classified as positive, 29.8% as negative, and 40.6% as neutral. Patient perception of observational strategies was consistently negative prior to 2007, with SI ranging from -13 to -14. However, beginning in 2008 there is a significant increase in sentiment, with SI ranging from +2 to +8. SI, stratified by year and terminology use, is shown in Figure 2.

### Patient Perception of Observational Management Strategies by Stratified Year of Diagnosis

#### 2002-2005 (n=31)

Patient perception at this time was overwhelmingly negative, with a SI of -13. Negative sentiment during this time period appears to be largely driven by patient perception that observational strategies can only be considered as a management option for older and sicker patients. There is also little reported physician support for WW or AS at this time to override negative sentiment. WW and AS are frequently described as “doing nothing,” which patients consider to be contrary to their own attitudes of PC treatment. Patients commented that although they realize WW or AS may be safe treatment options, many of them do not have the proper mindset to cope with the anxiety associated with frequent PSA testing and biopsies. Finally, patients comment that the lack of quantitative information regarding WW or AS makes it difficult to seriously consider these treatment options as compared to surgery or RT, for which there are ample resources for quantitative information regarding cure rates and risks of treatment-related toxicity.

Representative comments from 2002–2005 are summarized in Table 2.

#### 2006-2007 (n=43)

Overall SI over this time period remains negative (-14). Several factors contributing to negative patient sentiment about WW and AS during 2002-2005 remain prevalent over this time period, including: 1) anxiety associated with receiving “no treatment;” 2) perception that observational strategies can never be considered for younger patients; and, 3) physician reluctance to recommend WW or AS.

Beginning in 2006, there is an increased presence of female participants in ICs, who are involved in determining whether WW or AS would be a safe management strategy for their spouses. Patients discuss recent advances in surgical and RT treatments, such as robotic RP or proton beam therapy, and wonder if WW or AS should still be considered valid treatment options in the light of these advances. There are also new discussions during this time period about medico-legal issues surrounding WW or AS. In a few instances, attorneys or their representatives attempt to contact patients in these ICs to seek potential clients who were harmed by choosing WW or AS.

For the subset of patients who do agree to be managed with WW or AS, there is an emerging interest in complementary alternative medicine (CAM) or partaking in diet/exercise changes in order to feel as though they are actively “doing something.”

Representative comments from 2006–2007 are summarized in Table 3.

#### 2008-2009 (n=144)

SI increased to +8 over this time period, as compared to -14 in 2006-2007. The increase in sentiment is accompanied by: 1) an increased awareness and acceptance that quality-of-life (QoL) issues should be a major consideration when making PC treatment decisions; 2) statements by the American Urological Association (AUA) endorsing AS as a safe treatment option for men with low-risk PC; and, 3) increased participation in ICs by men being managed with AS who are willing to share their own “success stories.”

ICs at this time also demonstrate instances of disagreement between male PC patients, who are focused primarily on QoL factors, and their female partners, who are focused primarily on risk of PC progression. Many women compare PC to breast cancer, where aggressive treatments are recommended.

There are also a growing number of younger men participating in ICs who now express a willingness to consider AS, but who also express frustration with the difficulty in finding a physician who is willing to endorse AS. Other patients comment on the absence of public role models or physicians who advocate strongly for AS or who opted for AS themselves after being diagnosed with PC.

Representative comments from 2008–2009 are summarized in Table 4.

#### 2010-2012 (n=246)

There is a drop in sentiment after the rise observed in 2009 (+2 vs. +8). Although ICs at this time demonstrate an increase in patient acceptance of AS, there was growing concern that physicians will be unable to provide patients with unbiased treatment recommendations. Patients increasingly cite data from prospective trials and other studies that support AS. However, patients question whether treating physicians will ‘evolve’ to better appreciate AS. Patients relate that physicians often ‘scare’ patients into undergoing surgery or RT. Patients further comment that the information they receive during physician consultations is inadequate for fully addressing all the different PC treatment options.

Patients propose solutions for addressing barriers to AS selection as a PC management option, including: 1) creation of a distinct medical specialty that focuses on AS; 2) use of an AS-specific billing code; 3) increased availability of educational resources from trusted medical organizations for patients and families; and, 4) increased presence of public AS role models and advocates.

ICs at this time also demonstrate a growing recognition from patients that routine prostate biopsies recommended as part of AS protocols can also impact one’s QoL negatively. Recent recommendations by the United States Preventive Services Task Force (USPSTF) against PSA testing have been a source of confusion for patients given the central role of PSA testing in AS protocols.

Representative comments from 2010–2012 are summarized in Table 5.

## Discussion

Treatment decision-making for men with low-risk PC is a highly complex and individualized process [11]. Patient decisions are influenced by a variety of factors, including physician advice, patient perception of PC, QoL considerations, and advice from friends and family members [11–15]. A growing number of men now also utilize the internet and online support groups as an additional resource for seeking information and expressing opinions about the treatment decision-making process [16]. The current study builds upon the contributions from prior qualitative studies by collecting and evaluating anonymized, free-form data through NLP from a large number of ICs on the subject of WW and AS. The cohort of patient in the present analysis is distinct from patients included in prior qualitative studies. Unlike patients typically included in traditional interview or survey studies, there are no geographical restrictions limiting who can participate in ICs [17,18]. Thus, the cohort included in this study likely represents a more diverse group of individuals, including current or former patients, family members, and patient advocates, who can provide us with varied viewpoints [17,18]. Moreover, the anonymous nature of the internet allows for patients to discuss sensitive issues that they would not typically discuss with physicians [18].

Based on the ICs evaluated, patients are increasingly receptive to considering AS as a PC management strategy, but many question if physicians can provide unbiased treatment recommendations. Data to support this concern comes from previous studies which have demonstrated that the specialty of physicians seen prior to treatment significantly impacts the type of PC treatment received [19]. Given the complexity of issues surrounding each of the different PC treatment options, ICs also indicate that patients desire more detailed and quantitative information about PC treatment options than what is typically provided in a physician consultation. A solution to this problem proposed in ICs is increased availability of detailed educational materials and decision aids, such as pamphlets and podcasts, to address these issues.

A novel finding in this study was a patient desire for the creation of a new breed of specialists-“active surveillists”—who can advocate for AS and provide information regarding AS that is similar in detail to information available regarding RT and surgery. Access to such a specialty may lend further credibility to the use of AS a PC management, and may also alleviate patient/public concern about receiving biased information from providers about PC treatment. Perhaps this idea could be realized through the inclusion of a non-biased PC specialist, such as a medical oncologist or internist, to represent AS in multidisciplinary PC clinics. Given that our study and previous studies have indicated that use of CAM may increase adherence to an AS program for interested patients [15], an AS multidisciplinary clinic may be an opportunity to provide patients with safe and reliable information regarding CAM or lifestyle modification programs. The value of multidisciplinary clinics for PC has been previously described [20], and this model may enhance shared decision-making for AS [21].

The current study also provides insights regarding temporal trends in public sentiment regarding AS and WW. Based on the SIs identified in this study, it appears that sentiment has become more positive over time. Prior to 2007, the majority of ICs focused on WW, with predominantly negative sentiment. During this time period, WW and AS were regarded as management strategies for sick or elderly patients and the overall perception was that WW/AS protocols were “too amorphous” a concept, with insufficient quantitative data that patients could refer to, to quantify risks associated with WW/AS. Later, in 2009, the majority of ICs on observational strategies for PC emphasized AS, rather than WW, and sentiment was positive. In contrast to the ICs from earlier years, patients seem to recognize that AS is an individualized choice that may be appropriate for some younger or healthier men, and patients cite data from AS guidelines or clinical trials. Interestingly, public sentiment was highest in 2008-2009, and ICs at this time often refer to a statement by the AUA endorsing AS as a safe management option for men with low-risk PC. Therefore, sentiment regarding AS increased over time, and information from clinical trials and consensus statements appears to have influenced the improvement in sentiment.

Based on this study, another method for increasing patient acceptance of AS would be through an increased presence of public figures who have chosen AS as a primary management strategy for themselves. While there have been many instances of public figures with PC who have spoken publically about their decision and experience to undergo definitive treatment [22,23], there are few public role models managed with AS to whom patients can refer. Such a method would not only increase public awareness of AS, but may also legitimize AS as a safe management strategy for certain PC-cases.

Our study also highlights a potential for misuse of internet forums to disseminate inaccurate or biased information to influence treatment decisions. Uninformed patient accounts may mislead others and negatively impact treatment decision making. A potential solution for this may be for increased presence of online support groups that are sponsored by healthcare organizations or patient advocacy groups. Previous studies examining the impact of healthcare online communities on patient perception of their overall care and towards their healthcare organization have demonstrated a positive impact [24].

There are several limitations to this study that must be acknowledged. First, since our study is limited to internet users, comments in online support groups may not be generalizable to the entire population of PC patients and their family members. Patients utilizing the internet are generally thought to be younger and have a higher educational level than non-users [25]. Moreover, given the anonymous nature of these ICs, it is not possible to ascertain demographic data (age, race, socioeconomic status, etc.) unless a poster specifically mentions this data. The retrospective and anonymous nature of the content does not permit investigators to clarify material in the ICs, and it is possible that some patient concerns may be under- or over-represented in ICs and could be better communicated during semi-structured interviews. Finally, although our analysis did identify a large sample of ICs on this subject, it is possible that some ICs were not identified due to the search methodology employed by the NLP software. Thus, such analyses should be considered as complementary to, but not replacements for, traditional survey- and interview-based studies.

## Conclusion

These internet-based data provide novel insight into decisions regarding AS and WW and may be utilized by healthcare providers to help identify unmet PC patient needs related to AS. This study additionally demonstrates the potential utility of NLP to analyze internet content in order to provide insight into patient preferences, sentiment, and decision-making. Based on our findings, we recommend that future decision tools for AS include quantitative data regarding outcomes after AS, so that patients can make decisions with an amount of information that is more similar to the resources available regarding RT or surgery.
