# Correction: Identifying Barriers to Patient Acceptance of Active Surveillance: Content Analysis of Online Patient Communications

**DOI:** 10.1371/annotation/88e8da00-ddc9-4855-8d00-4490e1f3b4fd

**Published:** 2013-09-12

**Authors:** Mark V. Mishra, Michele Bennett, Armon Vincent, Olivia T. Lee, Costas D. Lallas, Edouard J. Trabulsi, Leonard G. Gomella, Adam P. Dicker, Timothy N. Showalter

The affiliations for authors Mark V. Mishra and Adam P. Dicker are incorrect. The correct affiliations can be found below.

Mark V. Mishra

Department of Radiation Oncology, Kimmel Cancer Center, Thomas Jefferson University, Philadelphia, Pennsylvania, United States of America

Adam P. Dicker

Department of Radiation Oncology, Kimmel Cancer Center, Thomas Jefferson University, Philadelphia, Pennsylvania, United States of America 

